# Full-length model of the human galectin-4 and insights into dynamics of inter-domain communication

**DOI:** 10.1038/srep33633

**Published:** 2016-09-19

**Authors:** Joane K. Rustiguel, Ricardo O. S. Soares, Steve P. Meisburger, Katherine M. Davis, Kristina L. Malzbender, Nozomi Ando, Marcelo Dias-Baruffi, Maria Cristina Nonato

**Affiliations:** 1 Laboratório de Cristalografia de Proteínas, Faculdade de Ciências Farmacêuticas de Ribeirão Preto, Universidade de São Paulo, SP, Brazil; 2 Department of Chemistry, Princeton University, Princeton, NJ, USA; 3 Departamento de Análises Clínicas, Toxicológicas e Bromatológicas, Faculdade de Ciências Farmacêuticas de Ribeirão Preto, Universidade de São Paulo, SP, Brazil

## Abstract

Galectins are proteins involved in diverse cellular contexts due to their capacity to decipher and respond to the information encoded by β-galactoside sugars. In particular, human galectin-4, normally expressed in the healthy gastrointestinal tract, displays differential expression in cancerous tissues and is considered a potential drug target for liver and lung cancer. Galectin-4 is a tandem-repeat galectin characterized by two carbohydrate recognition domains connected by a linker-peptide. Despite their relevance to cell function and pathogenesis, structural characterization of full-length tandem-repeat galectins has remained elusive. Here, we investigate galectin-4 using X-ray crystallography, small- and wide-angle X-ray scattering, molecular modelling, molecular dynamics simulations, and differential scanning fluorimetry assays and describe for the first time a structural model for human galectin-4. Our results provide insight into the structural role of the linker-peptide and shed light on the dynamic characteristics of the mechanism of carbohydrate recognition among tandem-repeat galectins.

Galectins are a family of glycan-binding proteins characterized by their affinity for β-galactosides and the presence of one or more structurally conserved carbohydrate recognition domains (CRDs)^1^. With fifteen members identified in vertebrates, galectins display diversity in ligand specificity and can be found in both intracellular and extracellular environments^2^,^3^. Notably, galectins have been shown to act as modulators of cell behaviour by regulating signalling processes as well as inflammatory and immune responses^4^. Galectins are promising candidates as diagnostic markers and novel drugs targets for a number of human diseases^4^,^5^.

To date, three subtypes of galectins have been identified, based on the number and structural arrangement of the CRDs: prototype, chimera and tandem-repeat^6^. While high-resolution structures of many full-length galectins remain elusive, crystallographic studies have revealed a significant structural similarity among CRDs. Common to most CRDs is a conserved β-sandwich fold with an overall jellyroll topology as well as a signature sequence for carbohydrate recognition^7^.

The tandem-repeat subtype of galectins contains two distinct CRDs (galectin-4N at the N-terminus and galectin-4C at the C-terminus) connected in a single polypeptide chain by a linker region^6^. Studies with tandem-repeat galectins have shown that the linker’s role, likely mediating the intramolecular interactions of CRDs, is associated with potency in inducing a specific biological response^8^,^9^,^10^,^11^,^12^,^13^. Other proposed roles for the linker region include protein-protein interactions, membrane insertion, and positioning the CRDs^10^,^11^,^13^.

Despite the importance of the linker, structural studies of galectins have thus far been limited to the individual CRDs or to engineered tandem-repeat galectins where the linker has been truncated. Furthermore, the anticipated flexibility of the linker and its susceptibility to proteolysis have made structural characterizations of full-length tandem-repeat galectins particularly challenging. In order to unravel the structural mechanisms that govern signalling modulation by tandem-repeat galectins, we chose human galectin-4 as our model of study. Galectin-4 belongs to the tandem-repeat category of galectins, together with galectins -6, -8, -9 and -12. Galectin-4 is largely expressed by intestinal epithelial cells and shows antagonist effects depending on the type of cancer.

Galectin-4 functions as a tumour suppressor of human colorectal and pancreatic cancer^14^,^15^,^16^. By contrast, in liver and lung cancer, the leading types of cancer that cause death worldwide, galectin-4 expression leads to increased metastasis and cancer progression^17^,^18^, suggesting its use as a promising target for drug development^5^. Here, we provide the first structural characterization of the full-length human galectin-4 using X-ray crystallography, small- and wide-angle X-ray scattering (SAXS/WAXS), molecular modelling, molecular dynamics simulations, and differential scanning fluorimetry assays. Our findings reveal that full-length galectin-4 folds as a compact structure and provide insight into the process by which the linker-peptide mediates recognition through correlated movements and transient interactions. These results shed light on the structural role of galectin-4’s linker-peptide and its biological function in this important class of proteins. Moreover, the generated knowledge and experimental tools described here can be exploited to investigate the role of galectin-4 under different pathological conditions.

## Results

### Protein production and thermal analysis of galectin-4, galectin-4N and galectin-4C

Galectin-4 is composed of 323 amino acids residues, which can be divided into an N-terminal domain (aa 1–150; galectin-4N), linker-peptide (aa 151–178) and C-terminal domain (aa 179–323; galectin-4C)^19^ (Supplementary Fig. S1). The full-length protein and its individual domains, galectin-4N and galectin-4C were cloned, overexpressed, and purified as described in the methods section. First, the folding stability of each construct was examined by differential scanning fluorimetry (Thermofluor), a methodology used to monitor protein unfolding. By measuring the fluorescence-probe intensity as a function of temperature, thermofluor assays allow for the comparison of melting temperatures (*T*_*m*_), transition profiles and thermal shift (Δ*T*_*m*_) values compared to the reference curves (obtained in buffer) at different conditions. Here, a positive Δ*T*_*m*_ indicates thermal stabilization induced by changes in the physicochemical environment.

**Figure 1:**
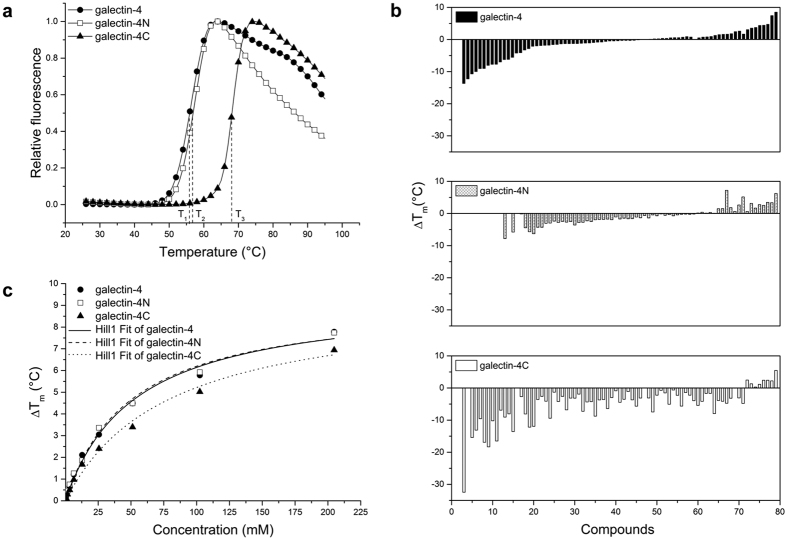
Thermofluor assays. (**a)** Normalized thermal denaturation curves for galectin-4, galectin-4N and galectin-4C. Measured apparent unfolding temperatures were 55.92 ± 0.05 °C for galectin-4, 56.8 ± 0.1 °C for galectin-4N and 68.12 ± 0.05 °C for galectin-4C. (**b**) Evaluation of thermal shift profile for galectin-4, galectin-4N and galectin-4C at different categories of additives. Bars show all additives that contribute to interpretable transitions with positive and/or negative thermal shift for the three proteins. Compounds and the respective thermal shift values are listed in Supplementary Table S1. (**c**) Thermal shift profile as function of lactose concentration.

Reference curves resulted in sigmoidal profiles with respective *T*_*m*_ values of 55.92 ± 0.05 °C for galectin-4, 56.8 ± 0.1 °C for galectin-4 N and 68.12 ± 0.05 °C for galectin-4C (Fig. 1a). The thermal behaviour of galectin-4 and its domains was also evaluated against the 94 additives from the Solubility & Stability Screen kit (Hampton Research) (Supplementary Table S1). Analysis of thermal shift (Δ*T*_*m*_) values in the presence of additives revealed that galectin-4C displays the largest Δ*T*_*m*_ values and the most distinctive behaviour under changes in the physicochemical environment (Fig. 1b). Lower Δ*T*_*m*_ values are observed for the full-length protein than its CRDs, suggesting that the galectin-4 gained stability due to the interaction between the CRDs.

The thermal shift of the three constructs was also evaluated in the presence of lactose, a low affinity β-galactoside ligand for galectin-4. A hyperbolic profile dependence on lactose concentration was observed, allowing for the estimation of saturating Δ*T*_*m*_ values of 9.4 ± 0.4 °C, 9.3 ± 0.4 °C and 9.3 ± 0.6 °C, for galectin-4, galectin-4N and galectin-4 C, respectively (Fig. 1c). Fitting of the apparent binding constant, *k*, for lactose yields similar values for galectin-4 and galectin-4 N of 53 ± 6 and 50 ± 5 mM, respectively, and a *k* of 78 ± 10 mM for galectin-4C. Apparent affinities obtained by thermofluor, which are proportional to the dissociation constants^20^, are in agreement with previous findings which described lactose as a weak ligand with galectin-4C displaying 1.5 times lower affinity than galectin-4N (1.3 mM and 1.9 mM for galectin-4N and galectin-4C, respectively^21^,^22^).

Additionally, melting curves for full-length galectin-4 were evaluated at different ionic strengths and pH values using the Solubility & Stability Screen 2 kit (Hampton Research). Although the *T*_*m*_ for full-length galectin-4 was lower with decreasing pHs, the melting curves consistently occurred in a single-domain protein denaturing event, suggesting that the global structure of galectin-4 remains stable as a compact unit over a wide range of conditions.

### Structural models for galectin-4N, galectin-4C and full-length galectin-4

**Figure 2:**
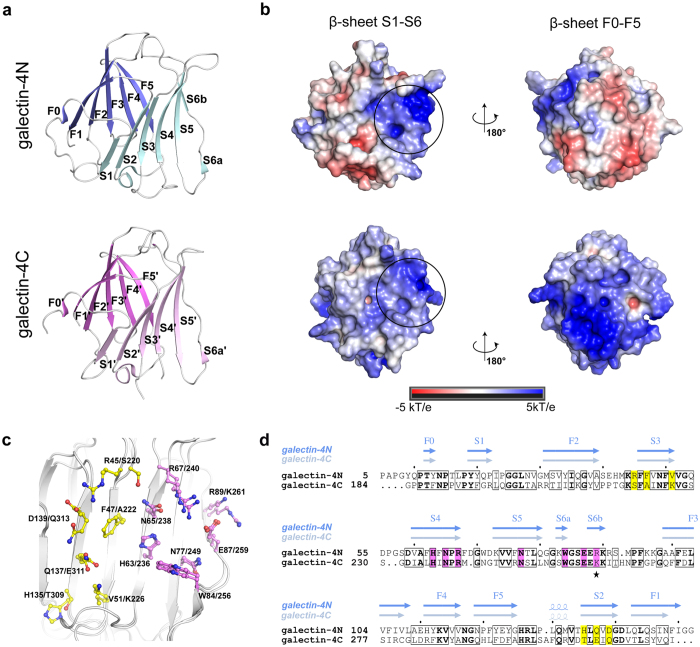
Crystal structures of galectin-4N and galectin-4C. (**a**) Overall β-sandwich fold of galectin-4N (blue) and galectin-4C (pink) structures. The antiparallel β-sheets are shown in blue (F0-F5) and cyan (S1-S6a/b) for galectin-4N, and pink (F0′-F5′) and light pink (S1′-S6a′) for galectin-4C. (**b**) Electrostatic potential surface for both the galectin-4N and galectin-4C structures. Front view (β-sheet S1-S6/S1′-S6′) and back view (β-sheet F0-F5/F0′-F5′). The circle marks the canonical binding site. (**c**) Canonical (pink) and extended (yellow) binding sites of galectin-4 domains. The main residues involved in binding interactions are represented as sticks. (**d)** Sequence alignment of galectin-4N and galectin-4C showing secondary structures elements. Marked in bold are the conserved residues. Highlighted in pink are the residues of canonical carbohydrate-binding site; the star is the only conservative substitution in the binding site residues between both domains. In yellow are the extended binding site residues.

To elucidate the full structural architecture of galectin-4, we solved the crystal structures of galectin-4N and galectin-4C at 1.48 Å and 1.78 Å resolution, respectively^23^,^24^ (Table 1). The final models for galectin-4N and galectin-4C are comprised of residues 5 to 152 and 184 to 323, respectively and share the same structural features previously described by Bum-Erdene and co-workers^21^,^22^. Both structures show the canonical β-sandwich fold arranged in a jellyroll topology, in which the monomer is formed by two antiparallel β-sheets, each composed of six (F0-F5/F0′-F5′ and S1-S6/S1′-S6′) β-strands (Fig. 2a).

Structural analysis of both galectin-4N and galectin-4C domains, which share a root mean square deviation (RMSD) of 1.2 Å between Cα atoms, reveal a large difference in charge distribution when the electrostatic potential surface is calculated at the physiological pH 7.4 (Fig. 2b). The galectin-4C surface charge distribution is mostly positive, whereas, the galectin-4N surface displays a more heterogeneous distribution with a positive region localized in the binding site.

The carbohydrate-binding site is located in a shallow pocket composed of residues present in the S4/S4′, S5/S5′ and S6/S6′ strands and the S5/S5′ adjacent loop. The residues involved are His63/236, Asn65/238, Arg67/240, Asn77/249, Trp84/256, Glu87/259 and Arg89/Lys261 in the galectin-4N/galectin-4C structures, respectively (Fig. 2c,d). The S2/S2′ and S3/S3′ strands, thought to contribute to the selectivity between galectin-4N and galectin-4C domains, form an extended cleft that permits interaction with different ligands. The main amino acid substitutions in the galectin-4N/galectin-4C structures are His135/Thr309, Gln137/Glu311 and Asp139/Gln313 for the S2/S2′ strand and Arg45/Ser220, Phe47/Ala222 and Val51/Lys226 for the S3/S3′ strand (Fig. 2c,d). Arg45 in the S3 strand from galectin-4N has been identified as the main residue to interact with a cholesterol sulphate ligand^25^ and to contribute weakly to lactose-3′-sulfate interaction^22^. Asn224 and Lys226 (S3′ strand), as well as Glu311 and Gln313 (S2′ strand) from galectin-4C have been shown to establish additional interactions with lacto-N-tetraose and lacto-N-neotetraose ligands^21^. Also in galectin-4C, Ser220 was identified as responsible for A-type saccharide preference^21^. Additional differences are observed in the loops between strands S3/S3′-S4/S4′ and S4/S4′-S5/S5′, where insertions are observed when comparing galectin-4N and galectin-4C amino acid sequences (Fig. 2d).

**Figure 3:**
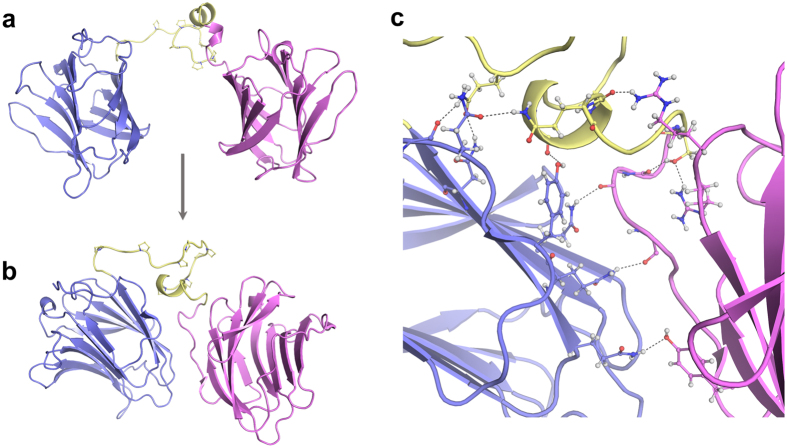
Model of full-length galectin-4. (**a**) Cartoon representation of the initial model for full-length protein (**b**) Overall fold of galectin-4 model after equilibrium dynamics and geometry optimization. (**c**) Representation of inter-domain interactions mediated by hydrogen bonds.

A structural model for full-length galectin-4 was obtained by combining molecular modelling and molecular dynamics (MD) simulations. First, *ab initio* prediction was used to generate different models of the linker-peptide. The best models, which share a compact structure and the presence of a short helix segment, were elected based on geometry and agreement between observed and predicted content in secondary structure. The linkers were combined as a single polypeptide chain with the X-ray structures of galectin-4N and galectin-4C, which were randomly arranged in relation to each other giving rise to six different starting models for full-length galectin-4. The model with the lowest potential energy (Fig. 3a) was submitted to a conformational refinement by MD. We began with a standard backbone-restrained solvation and thermalisation (2 ns) to achieve a pressure of 1 atm and a temperature of 37 °C (310 K) in the simulation box. A 30 ns production simulation was subsequently performed to ensure that the system reached and maintained proper equilibrium. The resulting trajectory was then analysed by principal component analysis (PCA), allowing us to select the lowest energy frame, which was designated as the starting point to all further rounds of MD simulations described in this work (Fig. 3b).

The galectin-4 model displays four antiparallel β-sheets connected by a linker-peptide that can be described as a proline-rich hinge followed by a short α-helix (amino acids 170–173) and an extended region (Fig. 3b). We observe a compact structure, having overall dimensions of 74 Å × 55 Å × 45 Å, in which the CRDs interact with each other and with the linker-peptide. These interactions are stabilized by 10 hydrogen bonds and 152 non-bonded contacts (Fig. 3c). The contact areas between interfaces were determined to be 465 Å^2^ (galectin-4N/linker), 349 Å^2^ (galectin-4N/galectin-4C) and 418 Å^2^ (linker/galectin-4 C).

### Solution conformation of human galectin-4

**Figure 4:**
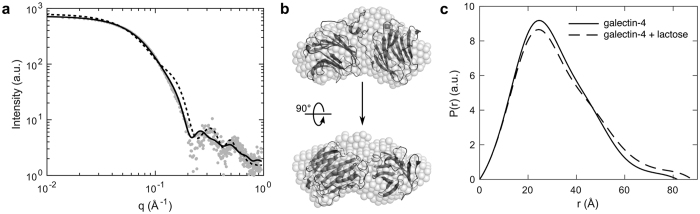
Solution conformation of full-length galectin-4 examined by X-ray scattering. (**a**) The experimental scattering of galectin-4 in the absence of ligand (gray) is well fit by the theoretical scattering of the full-length model in Fig. 3b (solid line), confirming that the two CRDs associate in solution. In contrast, a comparison of the experimental scattering to the theoretical scattering of the model found in Fig. 3a in which the CRDs are non-associating (dotted), shows a poor fit. (**b**) An *ab initio* shape reconstruction generated from ligand-free galectin-4 scattering data also shows good agreement with the full-length model. (**c**) Addition of lactose leads to a subtle expansion in the width of the pair-distance distribution function, *P*(*r*), and a slight increase in radius of gyration.

To evaluate the energy-minimized full-length galectin-4 model obtained by MD (Fig. 3b), the overall conformation of the protein was examined in solution by X-ray scattering, a technique that is ideally suited for probing ligand-induced conformational changes and for examining dynamic proteins that are challenging to crystallise. In-line size exclusion chromatography (SEC) was used to separate any mixtures as well as to ensure accurate background subtractions. Scattering was measured over a wide range of scattering angles on galectin-4 both in the absence of any ligands and in the presence of 30 mM lactose (Supplementary Fig. S2). For each sample, approximately 500 exposures were collected as the elution flowed directly into a continuous-flow cell. In each case, sample homogeneity was confirmed in the central region of the elution peak (Supplementary Fig. S2, blue regions) by singular value decomposition (SVD) and Guinier analysis (Supplementary Fig. S2)^26^, and thus, the scattering profiles within these regions were averaged (Fig. 4a, gray circles). A comparison of the experimental curve with the theoretical scattering of a model of galectin-4, in which the CRDs are non-associating (Fig. 3a, dotted curve) shows a poor fit, whereas a comparison with the theoretical scattering calculated from the full-length model described above (Fig. 3b, black curve) shows remarkable agreement. Consistent with this result, the *ab initio* shape reconstruction of galectin-4 derived from the SAXS data also suggests a compact conformation in which galectin-4N and galectin-4C are associated (Fig. 4b). Interestingly, the scattering of galectin-4 in the presence of lactose is nearly superimposable with that of ligand-free galectin-4. Only a subtle difference is apparent at low angles, corresponding to features at large length scales. Consistent with this, Guinier analysis yields slightly different radii of gyration for galectin-4 without and with lactose of 23.7 ± 0.1 Å and 24.9 ± 0.1 Å, respectively. The subtle expansion in the conformation upon addition of lactose is best visualized by an increase in the width of the pair-distance distribution function, *P*(*r*) (Fig. 4c).

### Molecular dynamics simulations

We performed molecular dynamics simulations of both galectin-4 and the galectin-4-lactose complex to investigate the behaviour of the protein in the presence and absence of a ligand. For each system, we performed four independent trajectories of 100 ns using different seeds (named MD 1, MD 2, MD 3 and MD 4). Analysis of the RMSD for backbone atoms showed that all simulations systems reached equilibrium before 100 ns (Supplementary Figs S3 and S4). Variations among MDs simulations showed that the apo structure adopts two main conformations: an “open” conformation with an average Rg of 23 Å and a “closed” conformation with an average Rg of 22 Å (Supplementary Fig. S3). The Rg histogram for MDs also revealed that in the protein-lactose complex, galectin-4 is stabilized in the “open” conformation (Supplementary Fig. S4).

Analyses of RMSD plots for each independent domain (Supplementary Figs S3 and S4) reveal that galectin-4C remained stable throughout the MD trajectory. Galectin-4N was shown to converge to similar structures sharing in average 1.4 Å deviation. Larger conformational fluctuations were observed in the linker-peptide, as expected for this type of disordered secondary structural element (Supplementary Figs S3 and S4).

### Inter-domain communication in galectin-4

**Figure 5:**
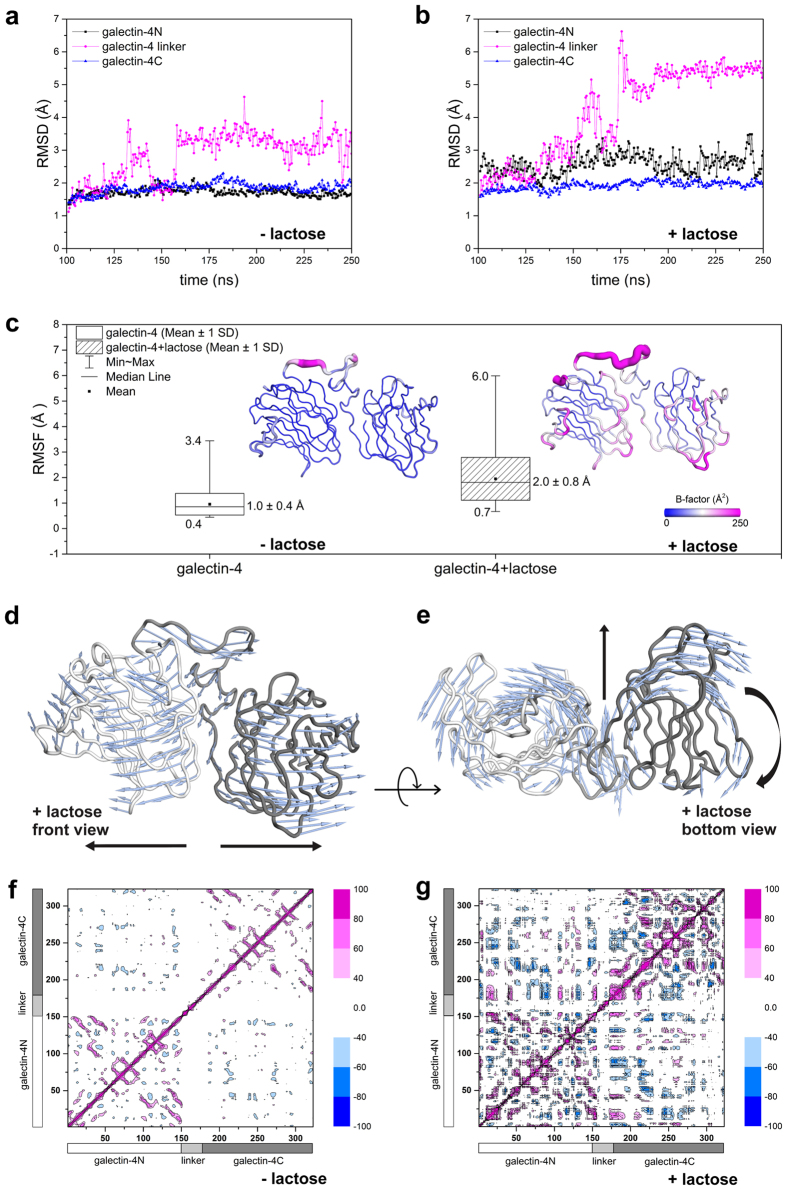
RMSD plots for molecular dynamics simulation with (+lactose) and without (−lactose) lactose, 150 ns trajectories. RMSD by domains structure (**a**) (−lactose) and (**b**) (+lactose). (**c)** RMSF box chart for MD simulation without and with lactose and cartoon putty representation of mobility through trajectory (inset); the blue-white-magenta scale calculated B-factor from 0 to 250 Å^2^. Porcupine plot of the first eigenvector generated through principal component analysis of the representative structure with lactose in (**d**) front view and (**e**) bottom view. The vectors, represented as blue arrows, show the tendency of movement. Plot of atomic correlations of MD without lactose (**f**) with lactose (**g**). The correlated movements are shown in pink and anticorrelated movements in blue scale bar. The bars indicate the portion of the graph relating to each domain, white for galectin-4N, light gray for linker and dark gray for galectin-4C.

To guarantee an investigation over a well-thermalized system we extended the MD 1 simulation to 250 ns and compared the 150 ns time interval, between 100 and 250 ns for both simulations (with and without lactose). RMSD plots (Fig. 5a,b) consistently showed differing galectin-4 behaviour in the absence and presence of lactose. In both cases, the linker-peptide generally demonstrated the highest deviation values, which are correlated with conformational changes associated to the full-length structure (Fig. 5a,b). Moreover, in the presence of the ligand, the galectin-4N domain showed a higher structural variability than galectin-4C.

For both MD simulations, we evaluated mobility using root mean square fluctuation (RMSF) box charts (Fig. 5c). The average RMSF was 1.0 ± 0.4 Å for galectin−4 and 2.0 ± 0.8 Å for the galectin-4-lactose system. Overall, the highest B-factors were in the galectin-4-lactose system, indicating greater flexibility than galectin-4 without lactose (Fig. 5c, inset). In both cases, the flexible regions were mainly found on the N-terminus, linker-peptide and regions between β-strands, with an emphasis on seven loops of galectin-4 (S3-S4, S5-S6, S3′-S4′, S4′-S5′, S5′-S6′, F4′-F5′ and F5′-S2′) and sixteen loops of galectin-4-lactose (F0-S1, F2-S3, S3-S4, S4-S5, S5-S6, S6-F3, S2-F1, F0′-S1′, F2′-S3′, S3′-S4′, S4′-S5′, S5′-S6′, S6′-F3′, F4′-F5′, F5′-S2′ and S2′-F1′).

This protein flexibility is related to the nature of intramolecular interactions. Hydrogen bond pairs with more than 10% occupancy were analysed between domains (Supplementary Table S2). For the MD simulation without lactose, we observed four H-bond pairs between galectin-4N/linker, five between galectin-4C/linker and four between galectin-4N/galectin-4C, of which, only five had greater than 50% occupancy. With lactose, there are seven H-bond pairs between galectin-4N/linker, nine between galectin-4C/linker and five between galectin-4N/galectin-4C, however only eight pairs interacted more than 50% of the time. Although the two MD simulations share only one H-bond pair, 148ASN(D22)-171HIS(ND1), eight common residues are involved in different H-bonding interactions. Moreover, a structural comparison between simulations at 250 ns revealed that the main interactions are non-bonded contacts, among which, many residues are the same in both systems.

Due to its more compact structure, the model without ligand showed larger interface areas than the galectin-4-lactose complex (Supplementary Fig. S5). The contact areas between surfaces in galectin-4 were determined to be 540 Å^2^ (galectin-4N/linker), 481 Å^2^ (galectin-4N/galectin-4C) and 334 Å^2^ (linker/galectin-4C). For the structure with lactose, these values were 325 Å^2^, 202 Å^2^ and 428 Å^2^, respectively. These interface areas suggest that in the first system the linker-peptide is shifted towards galectin-4N, while in the system with lactose it is shifted towards galectin-4C. The dynamic nature of the interface where the interaction are sustained by transient contacts, gives this region an intrinsic flexibility.

Principal component analysis (PCA) was used to estimate the primary domain motions (Fig. 5d,e). The results indicate that only a portion of the linker showed significant movement in the simulation without lactose. In contrast, both CRDs showed opposing rotational movements when in presence of lactose (Fig. 5d,e). According to the RMSD plot (Fig. 5b), the structural rearrangement in the linker is associated with a movement that pushes the CRDs in opposite directions (Fig. 5d,e).

Additionally, correlation plots showed that both structures, galectin-4 and galectin-4-lactose, have different structural correlation patterns (Fig. 5f,g). Galectin-4 mainly showed positive intra-domain correlations, with few anti-correlated movements between CRDs. Although the linker had shown high flexibility, its movement was not correlated with any domain (Fig. 5f). The galectin-4-lactose complex, in contrast, showed a larger number of positive and negative correlations (Fig. 5g), involving residues of all domains.

Despite movement, the low RMSD of each domain through trajectory (Fig. 5b) indicates low structural variability. Even so, galectin-4N and galectin-4C show long-range anti-correlated movements with respect to each other (Fig. 5g). The combination of these two behaviours reflects a correlated movement of rigid bodies mediated by the exchange of weak interactions with the linker.

## Discussion

It is well known that CRDs share a conserved β-sandwich fold and that there is a sequence signature for carbohydrate recognition and binding (Supplementary Fig. S1)^7^. However, one of the most notable properties about galectins and their CRDs is the meticulous way in which they discriminate among different glycans, resulting in a variable and complex biological response^27^,^28^.

Studies have demonstrated that the tandem-repeat galectins are more potent than galectins-1 and -3 in activating signalling in T cells and neutrophils^9^,^12^,^13^. In addition, they display a broad spectrum of biological activities as major signalling modulators both inside and outside the cell. This characteristic suggests that a combination of two distinct CRDs and a linker-peptide brings together chemical, structural and dynamic diversity able to impact on potency and on the plurality of carbohydrate-dependent events involved in their signalling ability and adhesive properties^10^.

The impact of tandem-repeat galectins on biological response has been associated with structural flexibility, relative orientation, and spacing between CRDs^9^. However, structural and dynamic characteristics of tandem-repeat galectins, including the type of interactions between CRDs and the linker-peptide, remain elusive and thus merit concentrated investigative efforts. However, despite the importance of this class of proteins in both physiological and pathological processes, the flexibility imposed by the linker and its susceptibility to proteolysis^29^ have made these studies very challenging.

As an important step toward assessing the underlying mechanisms that govern the function of tandem-repeat galectins acting on multiple targets, we presented for the first time a structural model of human galectin-4 based on a combination of theoretical and experimental approaches. The final model of galectin-4, constructed based on X-ray crystallography, molecular modelling and MD simulations and further supported by SAXS experiments, reveals that galectin-4 folds as a compact structure in which the CRDs interact both with each other and with the linker-peptide (Fig. 3b). The galectin-4 domains, galectin-4N, galectin-4C and the linker-peptide, were found to be mainly connected by weak (hydrogen and other non-bonded interactions) and transient contacts, revealing the dynamic nature of the interfacial interactions (Supplementary Table S2).

Experimental evidence for interaction between the CRDs was also observed when comparing the thermal denaturation profiles of the full-length galectin-4 with its independent domains (Fig. 1a). Although there was an 11 °C difference between the melting temperatures of the CRD domains, large enough to be distinguished if the unfolding process was characterized by sequential (non-cooperative) events of CRD domains, the profile for the melting curve obtained for full-length galectin-4 was consistent with a single-domain protein denaturing event (Fig. 1a). The same profile was observed when galectin-4 was submitted to different pH, ionic strengths and additives. This results reinforces the hypothesis that CRDs are not only associated under physiological conditions, but also remain together under diverse conditions, including those that mimic acidic extracellular microenvironments characteristic of tumour tissue^30^ in which the protein is often present.

Corroborating the idea of a compact structure, full-length galectin-4 was also shown to be more stable than its independent domains (Fig. 1b). In fact, a comparison of the melting curves of galectin-4, galectin-4N and galectin-4C allowed us to compare the behaviour of isolated CRDs with full-length galectin-4 and infer the individual contribution of each CRD for galectin-4 structure.

Differences between the galectin-4N and galectin-4C melting curves under the different conditions are notable (Fig. 1b, Supplementary Table S1) and can be explained as a consequence of variation in their chemical properties, i.e., number and charge distribution of amino acids among CRDs (Fig. 2b). Galectin-4C was shown to be more sensitive to changes in the chemical environment, displaying larger thermal shift (Δ*T*_*m*_) values, but it appears more stable than galectin-4N overall (Fig. 1b, Supplementary Table S1). In agreement, MD data shows that galectin-4C is more rigid (Fig. 5b), a requirement to compensate for increased thermal fluctuations. In contrast, the larger RMSD values observed during simulation reveal that galectin-4N can be more plastic (Fig. 5b), a characteristic that allows this domain to be more promiscuous in carbohydrate recognition and binding, as well as more potent in achieving a biological response.

Careful analysis of melting curves and thermal shift values under different chemical environments reveals that galectin-4 takes advantage of the stability of both domains to remain stable over a larger range of chemical conditions, i.e., the most stable domain governs the denaturation process of galectin-4 (Fig. 1b). This combined response is a reflection of its compact structure and of the ability of the linker-peptide to switch back and forth between CRDs that allows for transient interactions to stabilize the more susceptible domain (Supplementary Table S2).

The similarity between the hyperbolic profile dependence on lactose concentration for galectin-4 and galectin-4N indicates that the response for the full-length protein is governed by a single binding site with similar properties to those of galectin-4N domain (Fig. 1c). The lack of a clear evidence of the contribution of the galectin-4C binding site for full-length protein behaviour (Fig. 1c) can be explained as a result from the contribution of the linker, as observed in our MD simulations (Supplementary Fig. 5). Whether the cross talk between galectin-4N and galectin-4C has a positive or a negative impact on galectin-4C lactose recognition remains to be elucidated.

Thermofluor studies complemented by our MD data provide insight into protein flexibility under different conditions. These results demonstrated that the sequence variation among galectin-4-CRDs, although preserving the integrity of the CRD β-fold sandwich and sequence signature for carbohydrate recognition, enable CRDs to respond differently to a given chemical environment. Thus, physiologically, the CRDs not only work as agents of glycan recognition, but can also be considered biochemical sensors of the microenvironment important for adapting the lectin properties of galectin-4 to different conditions, and thereby assuring its biological impact in distinct physiological and pathological processes.

Different from the apo protein, the galectin-4-lactose complex is found stabilized in an open conformation, characterized by a hinge-bending motion (Fig. 5d,e) and a decrease in contact areas between domains (Supplementary Fig. S5). Consistent with our MD results (Supplementary Fig. S4), an increase in radius of gyration is observed by SAXS in the presence of lactose. Covariance analysis showed that the movement between linker and CRDs is directly correlated (Fig. 5g). Whereas, analysis of both RMSD and RMSF distributions demonstrates that both CRDs move as rigid bodies, without any significant intra-domain distortion or disruption of the carbohydrate-binding site (Fig. 5b,c).

Together, thermofluor, SAXS and MD analyses associate this lactose-stabilized, elbow-hinged switch in the full-length galectin-4 with a gain of thermal stability in each individual CRD domain (Fig. 1c) and flexibility (Fig. 5c). In another words, the enthalpy gain associated to lactose binding is compensated by an entropy loss within CRD domains and is correlated with an entropy gain in the full structure.

Our work also sheds light on the role of the linker-peptide as a key element in tandem-repeat galectins. In the galectin-4 model, the linker was observed to function as a molecular hinge that mediates the interaction between the CRDs (Fig. 3c), thanks to the high content of proline residues, 28.6%, that imposed severe restrictions in the conformation and movement of this region. In fact, a comparison among the five known tandem-repeat galectins and their isoforms reveals the existence of ten different linker-peptides characterized by high variability in length and amino acid distribution, but sharing a high content of proline residues (Supplementary Fig. S1). This feature affects the global structure of tandem-repeat galectins and in the manner in which the linker-peptide coordinates the movement and distance between CRDs. Thus, it is reasonable to predict that each member of the tandem-repeat galectin subfamily possesses a structural arrangement that depends on features of all individual domains. Galectin-4 and its homologue galectin-6, for example, share high sequence identity, but very distinct linker-peptides capable of offering unique structural and dynamic features for each protein, and in turn unique biological roles. Our model for galectin-4 provides the basis for further investigation.

Notably, all tandem-repeat galectin linker-peptides share proline-rich regions (PRRs). Besides their influence on protein structure and stability, PRRs are also described as binding domains^31^. In particular, they have a unique architecture which allows them to participate in molecular interactions that rely on multiple weak binding sites^31^. This architecture is characterized by restricted mobility, which reduces the unfavourable entropy loss of peptides upon binding. It is further influenced by the flat hydrophobic surface of prolines and the characteristics of the amide bond preceding proline, which make it a strong hydrogen bond acceptor. The unique architecture of PRRs can be particularly important in protein-protein and protein-nucleic acid interactions involved in intracellular signalling dependent on tandem-repeat galectins^4^. In particular, the continuous surface observed in galectin-4, as a consequence of its single domain arrangement, may favour protein-protein interactions including galectin-4 dimerization, as previously observed^25^. This is in contrast to a scenario in which the CRDs are flexible and move independently of each other.

In summary, a multi-technique approach has allowed us to investigate the structure of galectin-4 and its thermal and dynamic behaviours. Our results suggest that changes in the physicochemical environment have a direct effect on the ability to CRDs to reach different conformational states, and in turn modulate ligand recognition. The relative positions between the CRDs and the extent of cross talk between them depend on the structural features of linker-peptide, in an orchestrated mechanism of detection and response to a cellular stimulus.

## Methods

### Protein cloning, expression and purification

The human galectin-4 open reading frame (GenBank: CR536544.1), coding for amino acids 1–323, was amplified from a previously constructed plasmid encoding galectin-4 and was cloned into the *Eco*RI/*Xho*I site of the pET-28a (Novagen) modified vector, pET-28a-SUMO. This vector was designed to produce an N-terminal His-tagged SUMO fusion protein via the insertion of a carrier ubiquitin-like protein, SMT3 from *Saccharomyces cerevisiae* (UniProtKB/Swiss-prot: Q12306.1), between the *Nhe*I and *Bam*HI sites. DNA sequencing confirmed proper insertion of the galectin-4 gene fragment into the pET28a-SUMO vector. *Escherichia coli* Rosetta (DE3) cells (Novagen), transformed with the expression vector, were cultured in LB media containing 34 μg ml^−1^ chloramphenicol and 30 μg ml^−1^ kanamycin at 37 °C. Overproduction of recombinant galectin-4 was induced by adding 50 μM of isopropyl β-D-1-thiogalactopyranoside once the optical density OD_600_ reached 0.5. Growth continued for 24 h at 25 °C and 180 rev min^−1^. Cells were harvested by centrifugation at 10,000*g* for 10 minutes at 4 °C. The cell pellet was kept on ice and suspended in lysis buffer (50 mM monosodium phosphate pH 8.0, 600 mM NaCl, 14 mM β-mercaptoethanol and 1 tablet of EDTA-free SIGMA*FAST*^TM^ protease inhibitor cocktail). Cells were subsequently disrupted by ten 30 s, 10 W sonication pulses applied at 30 s intervals. The lysate was then clarified by centrifugation at 4 °C and 16,000 *g* for 30 minutes. The resulting supernatant was loaded onto a Ni-NTA column pre-equilibrated with buffer A (50 mM monosodium phosphate pH 8.0, 600 mM NaCl and 14 mM β-mercaptoethanol). The column was washed with a step gradient of 0 and 25 mM imidazole added to buffer A, at ten column volumes each. The His_6_-SUMO-galectin-4 fusion eluted with ten column volumes of buffer A plus 500 mM imidazole. Protein fractions were identified by their absorbance at 280 nm, pooled, concentrated using a 10 kDa cut-off centrifugal filter unit Amicon^®^ Ultra-15 (Millipore) and dialyzed against buffer A. The His_6_-tagged SUMO was cleaved by a ULP1 protease (Ubiquitin-like-specific Protease 1– EC 3.4.22.68) for 16 h at 8 °C. The sample was subsequently loaded onto a Ni-NTA resin column where galectin-4 was separated from ULP1 and SUMO through elution with buffer A plus 25 mM imidazole.

Galectin-4N (N-terminal domain from human galectin-4, residues 1–152)^23^ and galectin-4C (C-terminal domain from human galectin-4, residues 179–323)^24^ were cloned, expressed and purified as previously described. All three proteins were further submitted to size exclusion chromatography using a Superdex200 10/300 column (GE Healthcare) pre-equilibrated with 50 mM HEPES pH 7.2, 150 mM NaCl and 14 mM β-mercaptoethanol. Purity of the resultant fractions was analysed by SDS-PAGE stained with Coomassie Brilliant Blue.

### Thermofluor for galectin-4, galectin-4N and galectin-4C

Thermofluor was used to map the response to chemical environments of galectin-4 and its domains galectin-4N and galectin-4C. The experiments were conducted in an Mx3005P RT-PCR (Agilent Technologies) using SYPRO^®^ orange (492/610 nm) (Invitrogen) as a fluorescent probe to detect exposed hydrophobic regions of the proteins. Samples were filtered through 0.2 μm membranes (Millipore) and quantified at 280 nm based on the theoretical molar extinction coefficient. Analysis of the proteins’ thermal denaturation profiles were performed using a 96-well PCR plate (Agilent Technologies). The samples were heated from 25 °C to 95 °C at 1 °C/min and fluorescence measurements were taken. Thermal melting curves were processed as in the protocol described by Niesen and co-workers^32^, and the melting temperature was obtained using GraphPad Prism software (www.graphpad.com). For a comparison of the galectin-4, galectin-4N and galectin-4C denaturation profiles, we initiated a 20 μl reaction containing 10 μM protein in 25 mM HEPES pH 7.2, 75 mM NaCl, 7 mM β-mercaptoethanol and 5X SYPRO^®^ orange. In the same conditions, the behaviour of galectin-4 and its domains was assessed using the Solubility and Stability Screen (Hampton Research). Evaluation of the proteins’ behaviour in the presence of lactose was performed using serial dilution from a parent solution of 409.6 mM lactose. The behaviour of galectin-4 at different pHs and ionic strengths was assessed using the Solubility and Stability Screen 2^TM^ (Hampton Research). Here, we initiated a 20 μl reaction containing 2.8 μM protein in 2.5 mM HEPES pH 7.2, 7.5 mM NaCl, 0.7 mM β-mercaptoethanol and 5X SYPRO^®^ orange.

### Protein crystallisation, data collection and structural analysis

The galectin-4N and galectin-4C domains were crystallised as previously described^23^,^24^. Cryogenic X-ray diffraction data for galectin-4N and galectin-4C were collected at the Diamond Light Source (beamline I04-1) and the SRL/SLAC National Accelerator Laboratory (beamline BL12-2) respectively. The data were indexed with MOSFLM^33^ and reduction was performed with Scala^34^ and Aimless^35^ in the CCP4 suite^36^. The structure of galectin-4N was determined to 1.48 Å resolution using the previous solution^23^ as a search model in Phaser^37^, implemented in the PHENIX suite^38^. The galectin-4C structure was determined to 1.78 Å resolution as described^24^. Model building and refinement were performed with Coot^39^ and phenix.refine^38^. The quality of the final models was validated by MolProbity^40^, where Ramachandran statistics indicate that 98.1% of residues lie in the favoured regions with no outliers for both galectin-4N and galectin-4C final models. Figures were prepared with PyMOL^41^. Diffraction data and refinement statistics are shown in Table 1. Structures were analysed with Coot^39^, PyMol^41^ and PDBsum^42^.

### Modelling of linker-peptide and full-length galectin-4 construction

A sequence of 33 amino acid residues (from 153 to 185, QPLRPQGPPMMPPYPGPGHCHQQLNSLP TMEGP in which the underlined region corresponds to the linker-peptide) from galectin-4 was submitted to the ROBETTA server^43^ for *ab initio* structure prediction. Geometry idealization was performed for all resulting models using the *phenix.geometry_minimization* program^38^ and results were evaluated based on model quality with the MolProbity server. Crystallographic structures of galectin-4N and galectin-4C together with the top two linker-peptide models were used to build six different structures for galectin-4 using MODELLER v9.14^44^. Two steps of optimization were implemented in the model generating script, Variable Target Function Method (VTFM) and molecular dynamics simulations (MD). Conjugated gradient and simulated annealing were implemented between VTFM and MD routines. The resultant full-length models were also submitted to geometry idealization and analysed with the MolProbity server. As with the linker-peptide, the structures were compared and the best model was used for preliminary molecular dynamics simulations.

### Molecular dynamics simulations

Molecular dynamics simulations were carried out using the GROMACS package^45^ along with the AMBER99sb-ILDN force field parameters^46^. The temperature and pressure were set to 310 K and 1 atm, and controlled by the Nosé-Hoover^47^ and Parrinello-Rahman^48^ algorithms, respectively. The electrostatic interactions of each atom were treated with the Particle Mesh Ewald scheme and, like the non-bonded interactions (described by the Lennard-Jones potential), were limited to a cut-off radius of 1.0 nm. All water-bonded interactions were constrained by the SETTLE algorithm^49^, whereas LINCS^50^ was used to constrain the bonded interactions of the protein. The time step integration of the leap-frog algorithm was set to 2 fs.

### Galectin-4 starting MD model

The homology model was enclosed and centred in a dodecahedron box within a distance of 1.2 nm from the faces, and the system was explicitly solvated with the TIP3P water model^51^. The pH of each system was set indirectly to neutral according to the correspondent ionization states of the amino acids side-chains of the protein^52^. Therefore, the addition of counter ions Na^+^ and Cl^−^ was controlled to neutralize the protein charges and reach an ionic strength of 150 mM. In order to remove spurious molecular contacts, a steepest descent energy minimization was carried out, levelling the total potential energy of the system to a value smaller than 2000 kJ.mol^−1^.nm^−1^. Then a restriction potential of 1000 kJ.mol^−1^nm^2^ was applied to the *xyz* coordinates of the backbone amino acids for 2 ns in order to adjust the solvation layer on the surface of the protein. Afterwards, we produced a 30 ns trajectory, which allowed us to thermalize the system as well as adapt the protein structure to an aqueous environment. From the resulting trajectory, we performed principal component analysis using a covariance matrix and obtained the set of eigenvectors in order to sample its conformational space. We then selected the first and second projections, and fed the values to generate a trajectory on the average structure. The potential energy of the resulting model was minimized using the method of steepest descent.

### Galectin-4 molecular dynamics: equilibrium and production

The final galectin-4 model from MD energy minimization was submitted to four 100 ns trajectories in the absence and presence of the lactose ligand (β-D-galactopyranosyl-D-glucose), using different seeds. The starting complex model was built by three-dimensional superimposition of each CRD from galectin-4 with the CRDs from galectin-8 (PDB ID 3VKL). The side chains of residues from the binding site of galectin-4 were positioned as in galectin-8, complexed with lactose. Next, lactose was transferred into the binding site of galectin-4. The ligand was built and parameterized with the Glycam^53^ server^54^. We performed the solvation, energy minimization and restriction steps in the same way as described above for the protein model. The resulting structure and topology files were converted to the GROMACS notation with *acpype*^55^ and the runs were analysed by GROMACS tools, Bio3D^56^, VMD^57^ and Pymol^41^. Secondary structure was assessed with PROMOTIF program^58^ implemented in PDBsum analysis^59^.

### X-ray Scattering of full-length galectin-4

X-ray scattering measurements were performed at the G1 Station of the Cornell High Energy Synchrotron Source (CHESS) using 11.75 keV X-rays with a flux of 10^11^ photons per second at a beam size of 250 × 480 μm^2^. Small-angle and wide-angle X-ray scattering (SAXS/WAXS) images were collected simultaneously on two photon-counting detectors (Pilatus 100K) at sample-to-detector distances of 1.47 m and 0.42 m respectively. The SAXS detector covered a *q*-range of 0.014 to 0.336 Å^−1^, and the WAXS detector covered a *q-*range of 0.338 to 0.960 Å^−1^, where *q* is the momentum transfer, defined as *q* = (4π/λ)sin(2θ/2), where λ is the X-ray wavelength and 2θ is the scattering angle. Samples were passed continuously through an *in vacuo* X-ray sample cell^60^ via an in-line size exclusion column (GE Superdex 200 5/15GL) operated by a room-temperature GE Äkta Purifier using a flow rate of 0.075 ml min^−1^. The column was pre-equilibrated with the running buffer, consisting of 50 mM HEPES pH 7.2, 140 mM NaCl, and 9 mM DTT (−lactose), or the same buffer with 30 mM lactose added (+lactose). Protein samples were injected into a 50 μL loop at a concentration of 22.6 mg ml^−1^ (+lactose) and 20 mg ml^−1^, (−lactose). Approximately 500 eight-second exposures were collected per sample. Images were integrated and normalized by the incident X-ray intensity as measured by an N_2_-filled ion chamber located after the beam-defining slits. Data were processed and analysed following established protocols^61^ using the ATSAS suite of programs^62^ and custom code written in MATLAB. Predicted SAXS profiles were calculated using CRYSOL^63^ with maximum order of harmonics equal to 35 and Fibonacci grid of order 18. The SAXS and WAXS regions were merged prior to pair distance distribution analysis in GNOM^64^. *Ab initio* shape reconstructions were performed in GASBOR^65^. 10 models were generated with 323 dummy residues, and subsequently aligned and averaged in DAMAVER^66^. The final, most probable model had a normalized spatial discrepancy (NSD) of 1.07 with a standard deviation of 0.03.

## Additional Information

**Accession codes:** Atomic coordinates and structure factors have been deposited in the Protein Data Bank under accession codes 4XZP (galectin-4N) and 5CBL (galectin-4C).

**How to cite this article**: Rustiguel, J. K. *et al*. Full-length model of the human galectin-4 and insights into dynamics of inter-domain communication. *Sci. Rep.* **6**, 33633; doi: 10.1038/srep33633 (2016).

## Supplementary Material

Supplementary Information

## Figures and Tables

**Table 1:** Data collection and refinement statistics.

	galectin-4N
**Data collection**	
Space group	P6_1_22
Cell dimensions	
*a, b, c* (Å)	72.55, 72.55, 110.30
* *α, β, γ (°)	90, 90, 120
* *Resolution (Å)	31.73–1.48(1.56–1.48)
* R*_*sym*_	0.056(0.543)
* <I/σI>*	23.5(4.8)
* *Completeness (%)	100.0(100.0)
* *Redundancy	11.9(12.3)
* *No. total reflections	350,098(51,170)
* *No. unique reflections	29,321(4,172)
Refinement	
* *Resolution (Å)	1.48
* R*_*work*_/*R*_*free*_	15.0/18.4
No. atoms	
* *Protein	1231
* *Ligand/ion	1
* *Water	174
*B-*factors	
* *Protein	22.90
* *Ligand/ion	12.50
* *water	35.0
r.m.s. deviations	
* *Bond lengths (Å)	0.006
* *Bond angles (°)	1.11

Values in parentheses are for highest-resolution shell. Each dataset was collected from a single crystal.

## References

[b1] Barondes S. H., Cooper D. N., Gitt M. A. & Leffler H. Galectins. Structure and function of a large family of animal lectins. J Biol Chem 269, 20807–20810 (1994). 8063692

[b2] Hughes R. C. Secretion of the galectin family of mammalian carbohydrate-binding proteins. Biochim Biophys Acta 1473, 172–185 (1999). 10580137 10.1016/s0304-4165(99)00177-4

[b3] Leffler H., Carlsson S., Hedlund M., Qian Y. & Poirier F. Introduction to galectins. Glycoconj J 19, 433–440 (2004). 14758066 10.1023/B:GLYC.0000014072.34840.04

[b4] Compagno D. . Galectins: major signaling modulators inside and outside the cell. Curr Mol Med 14, 630–651 (2014). 24894174 10.2174/1566524014666140603101953

[b5] Ebrahim A. H. . Galectins in cancer: carcinogenesis, diagnosis and therapy. Ann Transl Med 2, 88 (2014). 25405163 10.3978/j.issn.2305-5839.2014.09.12 PMC4205868

[b6] Hirabayashi J. & Kasai K. The family of metazoan metal-independent beta-galactoside-binding lectins: structure, function and molecular evolution. Glycobiology 3, 297–304 (1993). 8400545 10.1093/glycob/3.4.297

[b7] López-Lucendo M. F. . Growth-regulatory human galectin-1: crystallographic characterisation of the structural changes induced by single-site mutations and their impact on the thermodynamics of ligand binding. J Mol Biol 343, 957–970 (2004). 15476813 10.1016/j.jmb.2004.08.078

[b8] Kashio Y. . Galectin-9 induces apoptosis through the calcium-calpain-caspase-1 pathway. J Immunol 170, 3631–3636 (2003). 12646627 10.4049/jimmunol.170.7.3631

[b9] Bi S., Earl L. A., Jacobs L. & Baum L. G. Structural features of galectin-9 and galectin-1 that determine distinct T cell death pathways. J Biol Chem 283, 12248–12258 (2008). 18258591 10.1074/jbc.M800523200 PMC2431002

[b10] Levy Y. . It depends on the hinge: a structure-functional analysis of galectin-8, a tandem-repeat type lectin. Glycobiology 16, 463–476 (2006). 16501058 10.1093/glycob/cwj097

[b11] André S., Wang G. N., Gabius H. J. & Murphy P. V. Combining glycocluster synthesis with protein engineering: an approach to probe into the significance of linker length in a tandem-repeat-type lectin (galectin-4). Carbohydr Res 389, 25–38 (2014). 24698724 10.1016/j.carres.2013.12.024

[b12] Earl L. A., Bi S. & Baum L. G. Galectin multimerization and lattice formation are regulated by linker region structure. Glycobiology 21, 6–12 (2011). 20864568 10.1093/glycob/cwq144 PMC2998985

[b13] Troncoso M. F., Elola M. T., Croci D. O. & Rabinovich G. A. Integrating structure and function of ‘tandem-repeat’ galectins. Front Biosci (Schol Ed) 4, 864–887 (2012). 22202096 10.2741/s305

[b14] Kim S. W. . Abrogation of galectin-4 expression promotes tumorigenesis in colorectal cancer. Cell Oncol (Dordr) 36, 169–178 (2013). 23378274 10.1007/s13402-013-0124-x PMC13012673

[b15] Belo A. I., van der Sar A. M., Tefsen B. & van Die I. Galectin-4 reduces migration and metastasis formation of pancreatic cancer cells. PLoS One 8, e65957 (2013). 23824659 10.1371/journal.pone.0065957 PMC3688853

[b16] Satelli A., Rao P. S., Thirumala S. & Rao U. S. Galectin-4 functions as a tumor suppressor of human colorectal cancer. Int J Cancer 129, 799–809 (2011). 21064109 10.1002/ijc.25750 PMC3071872

[b17] Hayashi T. . Galectin-4, a novel predictor for lymph node metastasis in lung adenocarcinoma. PLoS One 8, e81883 (2013). 24339976 10.1371/journal.pone.0081883 PMC3858289

[b18] Kondoh N. . Identification and characterization of genes associated with human hepatocellular carcinogenesis. Cancer Res 59, 4990–4996 (1999). 10519413

[b19] Huflejt M. E., Jordan E. T., Gitt M. A., Barondes S. H. & Leffler H. Strikingly different localization of galectin-3 and galectin-4 in human colon adenocarcinoma T84 cells. Galectin-4 is localized at sites of cell adhesion. J Biol Chem 272, 14294–14303 (1997). 9162064 10.1074/jbc.272.22.14294

[b20] Matulis D., Kranz J. K., Salemme F. R. & Todd M. J. Thermodynamic stability of carbonic anhydrase: measurements of binding affinity and stoichiometry using ThermoFluor. Biochemistry 44, 5258–5266 (2005). 15794662 10.1021/bi048135v

[b21] Bum-Erdene K., Leffler H., Nilsson U. J. & Blanchard H. Structural characterization of human galectin-4C-terminal domain: elucidating the molecular basis for recognition of glycosphingolipids, sulfated saccharides and blood group antigens. FEBS J 282, 3348–3367 (2015). 26077389 10.1111/febs.13348

[b22] Bum-Erdene K., Leffler H., Nilsson U. J. & Blanchard H. Structural characterisation of human galectin-4N-terminal carbohydrate recognition domain in complex with glycerol, lactose, 3′-sulfo-lactose, and 2′-fucosyllactose. Sci Rep 6, 20289 (2016). 26828567 10.1038/srep20289 PMC4734333

[b23] Zimbardi A. L., Pinheiro M. P., Dias-Baruffi M. & Nonato M. C. Cloning, expression, purification, crystallization and preliminary X-ray diffraction analysis of the N-terminal carbohydrate-recognition domain of human galectin-4. Acta Crystallogr Sect F Struct Biol Cryst Commun 66, 542–545 (2010). 10.1107/S1744309110010778 PMC2864688 20445255

[b24] Rustiguel J. K., Kumagai P. S., Dias-Baruffi M., Costa-Filho A. J. & Nonato M. C. Recombinant expression, purification and preliminary biophysical and structural studies of C-terminal carbohydrate recognition domain from human galectin-4. Protein Expr Purif 118, 39–48 (2016). 26432949 10.1016/j.pep.2015.09.026

[b25] Ideo H., Seko A. & Yamashita K. Recognition mechanism of galectin-4 for cholesterol 3-sulfate. J Biol Chem 282, 21081–21089 (2007). 17545668 10.1074/jbc.M703770200

[b26] Fagherazzi G. Small angle X-ray scattering edited by Glatter O. & Kratky O.. Acta Crystallographica Section A 39, 500 (1983).

[b27] Di Lella S. . When galectins recognize glycans: from biochemistry to physiology and back again. Biochemistry 50, 7842–7857 (2011). 21848324 10.1021/bi201121m PMC3429939

[b28] Rabinovich G. A., Toscano M. A., Jackson S. S. & Vasta G. R. Functions of cell surface galectin-glycoprotein lattices. Curr Opin Struct Biol 17, 513–520 (2007). 17950594 10.1016/j.sbi.2007.09.002 PMC2100406

[b29] Yoshida H. . X-ray structure of a protease-resistant mutant form of human galectin-8 with two carbohydrate recognition domains. FEBS J 279, 3937–3951 (2012). 22913484 10.1111/j.1742-4658.2012.08753.x

[b30] Kato Y. . Acidic extracellular microenvironment and cancer. Cancer Cell Int 13, 89 (2013). 24004445 10.1186/1475-2867-13-89 PMC3849184

[b31] van Weelden S., van Hellemond J., Opperdoes F. & Tielens A. New functions for parts of the Krebs cycle in procyclic *Trypanosoma brucei*, a cycle not operating as a cycle. Journal of Biological Chemistry 280, 12451–12460 (2005). 15647263 10.1074/jbc.M412447200

[b32] Niesen F. H., Berglund H. & Vedadi M. The use of differential scanning fluorimetry to detect ligand interactions that promote protein stability. Nat Protoc 2, 2212–2221 (2007). 17853878 10.1038/nprot.2007.321

[b33] Battye T. G., Kontogiannis L., Johnson O., Powell H. R. & Leslie A. G. iMOSFLM: a new graphical interface for diffraction-image processing with MOSFLM. Acta Crystallogr D Biol Crystallogr 67, 271–281 (2011). 21460445 10.1107/S0907444910048675 PMC3069742

[b34] Evans P. Scaling and assessment of data quality. Acta Crystallogr D Biol Crystallogr 62, 72–82 (2006). 16369096 10.1107/S0907444905036693

[b35] Evans P. R. & Murshudov G. N. How good are my data and what is the resolution? Acta Crystallogr D Biol Crystallogr 69, 1204–1214 (2013). 23793146 10.1107/S0907444913000061 PMC3689523

[b36] Winn M. D. . Overview of the CCP4 suite and current developments. Acta Crystallogr D Biol Crystallogr 67, 235–242 (2011). 21460441 10.1107/S0907444910045749 PMC3069738

[b37] McCoy A. J. . Phaser crystallographic software. J Appl Crystallogr 40, 658–674 (2007). 19461840 10.1107/S0021889807021206 PMC2483472

[b38] Adams P. D. . PHENIX: a comprehensive Python-based system for macromolecular structure solution. Acta Crystallogr D Biol Crystallogr 66, 213–221 (2010). 20124702 10.1107/S0907444909052925 PMC2815670

[b39] Emsley P., Lohkamp B., Scott W. G. & Cowtan K. Features and development of Coot. Acta Crystallogr D Biol Crystallogr 66, 486–501 (2010). 20383002 10.1107/S0907444910007493 PMC2852313

[b40] Chen V. B. . MolProbity: all-atom structure validation for macromolecular crystallography. Acta Crystallogr D Biol Crystallogr 66, 12–21 (2010). 20057044 10.1107/S0907444909042073 PMC2803126

[b41] DeLano W. L. Use of PYMOL as a communications tool for molecular science. Abstracts of Papers of the American Chemical Society 228, U313–U314 (2004).

[b42] Laskowski R. A. . PDBsum: a Web-based database of summaries and analyses of all PDB structures. Trends Biochem Sci 22, 488–490 (1997). 9433130 10.1016/s0968-0004(97)01140-7

[b43] Kim D. E., Chivian D. & Baker D. Protein structure prediction and analysis using the Robetta server. Nucleic Acids Res 32, W526–531 (2004). 15215442 10.1093/nar/gkh468 PMC441606

[b44] Sali A. & Blundell T. L. Comparative protein modelling by satisfaction of spatial restraints. J Mol Biol 234, 779–815 (1993). 8254673 10.1006/jmbi.1993.1626

[b45] Pronk S. . GROMACS 4.5: a high-throughput and highly parallel open source molecular simulation toolkit. Bioinformatics 29, 845–854 (2013). 23407358 10.1093/bioinformatics/btt055 PMC3605599

[b46] Lindorff-Larsen K. . Improved side-chain torsion potentials for the Amber ff99SB protein force field. Proteins 78, 1950–1958 (2010). 20408171 10.1002/prot.22711 PMC2970904

[b47] Hoover W. G. Canonical dynamics: Equilibrium phase-space distributions. Phys Rev A Gen Phys 31, 1695–1697 (1985). 9895674 10.1103/physreva.31.1695

[b48] Parrinello M. & Rahman A. Polymorphic transitions in single crystals: A new molecular dynamics method. Journal of Applied Physics 52 (1981).

[b49] Miyamoto S. & Kollman P. A. Settle: An analytical version of the SHAKE and RATTLE algorithm for rigid water models. Journal of Computational Chemistry 13, 952–962 (1992).

[b50] Hess B. P-LINCS: A parallel linear constraint solver for molecular simulation. J Chem Theory Comput 4, 116–122 (2008). 26619985 10.1021/ct700200b

[b51] Jorgensen W. L., Chandrasekhar J., Madura J. D., Impey R. W. & Klein M. L. Comparison of simple potential functions for simulating liquid water. Journal of Chemical Physics 79, 926–935 (1983).

[b52] Thurlkill R. L., Grimsley G. R., Scholtz J. M. & Pace C. N. pK values of the ionizable groups of proteins. Protein Sci 15, 1214–1218 (2006). 16597822 10.1110/ps.051840806 PMC2242523

[b53] Kirschner K. N., Lins R. D., Maass A. & Soares T. A. A glycam-based force field for simulations of lipopolysaccharide membranes: parametrization and validation. J Chem Theory Comput 8, 4719–4731 (2012). 26605626 10.1021/ct300534j

[b54] Group., W. *GLYCAM Web*, http://glycam.org/ (2005–2015).

[b55] Sousa da Silva A. W. & Vranken W. F. ACPYPE - AnteChamber PYthon Parser interfacE. BMC Res Notes 5, 367 (2012). 22824207 10.1186/1756-0500-5-367 PMC3461484

[b56] Grant B. J., Rodrigues A. P., ElSawy K. M., McCammon J. A. & Caves L. S. Bio3d: an R package for the comparative analysis of protein structures. Bioinformatics 22, 2695–2696 (2006). 16940322 10.1093/bioinformatics/btl461

[b57] Humphrey W., Dalke A. & Schulten K. VMD: visual molecular dynamics. J Mol Graph 14, 33–38, 27–38 (1996). 8744570 10.1016/0263-7855(96)00018-5

[b58] Hutchinson E. G. & Thornton J. M. PROMOTIF–a program to identify and analyze structural motifs in proteins. Protein Sci 5, 212–220 (1996). 8745398 10.1002/pro.5560050204 PMC2143354

[b59] de Beer T. A., Berka K., Thornton J. M. & Laskowski R. A. PDBsum additions. Nucleic Acids Res 42, D292–296 (2014). 24153109 10.1093/nar/gkt940 PMC3965036

[b60] Nielsen S. S., Møller M. & Gillilan R. E. High-throughput biological small-angle X-ray scattering with a robotically loaded capillary cell. J Appl Crystallogr 45, 213–223 (2012). 22509071 10.1107/S0021889812000957 PMC3325496

[b61] Skou S., Gillilan R. E. & Ando N. Synchrotron-based small-angle X-ray scattering of proteins in solution. Nat Protoc 9, 1727–1739 (2014). 24967622 10.1038/nprot.2014.116 PMC4472361

[b62] Petoukhov M. V. . New developments in the ATSAS program package for small-angle scattering data analysis. J Appl Crystallogr 45, 342–350 (2012). 25484842 10.1107/S0021889812007662 PMC4233345

[b63] Svergun D., Barberato C. & Koch M. CRYSOL - A program to evaluate x-ray solution scattering of biological macromolecules from atomic coordinates. Journal of Applied Crystallography 28, 768–773 (1995).

[b64] Svergun D. Determination of the regularization parameter in indirect-transform methods using perceptual criteria. Journal of Applied Crystallography 25, 495–503 (1992).

[b65] Svergun D. I., Petoukhov M. V. & Koch M. H. Determination of domain structure of proteins from X-ray solution scattering. Biophys J 80, 2946–2953 (2001). 11371467 10.1016/S0006-3495(01)76260-1 PMC1301478

[b66] Volkov V. V. & Svergun D. I. Uniqueness of *ab initio* shape determination in small-angle scattering. Journal of Applied Crystallography 36, 860–864 (2003). 10.1107/S0021889809000338 PMC5023043 27630371

