# Supplementary material for: Full-length model of the human galectin-4 and insights into dynamics of inter-domain communication
Source: Sci Rep. 2016 Sep 19;6:33633. doi: 10.1038/srep33633 (PMC5027518; doi:10.1038/srep33633)
Supplement: Supplementary Information [file srep33633-s1.pdf]

Supplementary information

Full-length model of the human galectin-4 and insights into dynamics of inter-domain communication

Joane K Rustiguel[1], Ricardo O S Soares[1], Steve P Meisburger[2], Katherine M Davis[2], Kristina L Malzbender[2], Nozomi Ando[2], Marcelo Dias-Baruffi[3], & M. Cristina Nonato[1*]

[1]Laboratório de Cristalografia de Proteínas, Faculdade de Ciências Farmacêuticas de Ribeirão Preto, Universidade de São Paulo, SP, Brazil

[2] Department of Chemistry, Princeton University, Princeton, NJ, USA

[3] Departamento de Análises Clínicas, Toxicológicas e Bromatológicas, Faculdade de Ciências Farmacêuticas de Ribeirão Preto, Universidade de São Paulo, SP, Brazil

[*]corresponding author cristy@fcfrp.usp.br

Supplementary Table S1

Solubility and stability screen compounds.

Description of compounds from Solubility and Stability screen kit (Hampton Research) used in the thermofluor analysis of galectin-4, galectin-4N and galectin-4C with the respective thermal shift values or transition curve profile.

				ΔTm (°C)		
		Category	***Compound description***	***galectin-4***	***galectin-4N***	***galectin-4C***
1	REF	water control	reference	0.0	0.0	0.0
2	A2	precipitate control	Trichloroacetic acid	-	-	-
3	D6	metal	50 mM Cadmium chloride hydrate/50 mM Cobalt(II) chloride hexahydrate	-13.66	-	-32.44
4	E9	ionic liquid	12.5% w/v 1-Butyl-3-methylimidazolium chloride	-12.24	-	-
5	C6	linker	250 mM Ethylenediamine dihydrochloride	-10.69	-	-15.41
6	A11	amino acid/derivative	250 mM L-Argininamide dihydrochloride	-9.96	-	-13.12
7	D4	chelator	25 mM Ethylenediaminetetraacetic acid disodium salt dihydrate	-9.04	-	-9.56
8	E10	ionic liquid	12.5% w/v Ethylammonium nitrate	-9.00	-	-16.89
9	C2	polyamine	250 mM Spermidine	-8.02	-	-18.32
10	E6	ionic liquid	12.5% w/v Tetraethylammonium bromide	-7.71	-	-10.2
11	F11	salt	125 mM Sodium p-toluenesulfonate	-7.69	-	-16.5
12	E8	ionic liquid	12.5% w/v 1-Ethyl-3-methylimidazolium acetate	-6.97	-	-6.9
13	D3	inhibitor	2.5% w/v Benzamidine hydrochloride	-6.23	-7.8	-9.05
14	D11	non detergent	400 mM Non Detergent Sulfobetaine 256 (NDSB-256)	-6.16	-	-8.05
15	F2	salt	250 mM Potassium thiocyanate	-5.54	-5.8	-13.55
16	H2	polyol	12.5% v/v Pentaerythritol ethoxylate (15/4 EO/OH)	-4.2	-	-
17	C3	linker	250 mM 5-Aminovaleric acid	-4.09	-0.15	-2.67
18	C7	chaotrope	250 mMGuanidine hydrochloride	-3.26	-4.45	-8.09
19	F10	salt	125 mM Sodium benzenesulfonate	-2.82	-5.69	-12.18
20	C1	polyamine	250 mM Spermine tetrahydrochloride	-2.09	-6.3	-11.93
21	G11	polyol	2.5% w/v Polyethylene glycol monomethyl ether 750	-1.98	-4.3	-3.57

22	D10	non detergent	500 mM Non Detergent Sulfobetaine 221 (NDSB-221)	-1.88	-4.3	-2.6
23	H3	polyol	5% w/v 1,2-Propanediol	-1.8	-3.07	-4.11
24	F6	salt	250 mM Lithium nitrate	-1.75	-2.98	-9.41
25	B1	peptide	250 mM Gly-gly	-1.57	-2.39	-1.3
26	C9	chaotrope	250 mM N-Methylurea	-1.51	-2.8	-4.18
27	B2	peptide	100 mM Gly-gly-gly	-1.33	-2.47	-2.6
28	E12	salt	250 mM Ammonium chloride	-1.28	-2.71	-6.81
29	E1	organic acid	250 mM Acetamide	-1.27	-2.51	-3.13
30	G9	polyol	5% v/v Polyethylene glycol 200	-1.26	-3.6	-3.16
31	B12	osmolyte	5% v/v Triethylene glycol	-1.25	-2.83	-1.89
32	D5	metal	50 mM Magnesium chloride hexahydrate/50 mM Calcium chloride dihydrate	-1.16	-2.74	-7.23
33	C10	chaotrope	100 mM N-Ethylurea	-1.11	-2.28	-4.34
34	G10	polyol	2.5% w/v Polyethylene glycol monomethyl ether 550	-1.06	-2.5	-4.24
35	G5	salt	500 mM Sodium bromide	-0.92	-1.96	-8.76
36	E7	ionic liquid	12.5% w/v Cholin acetate	-0.81	-1.86	-3.72
37	C8	chaotrope	250 mM Urea	-0.77	-1.83	-3.57
38	B3	peptide	2.5% w/v Tryptone	-0.56	-1.75	-6.38
39	G8	polyol	5% v/v Ethylene glycol	-0.44	-1.94	-2.96
40	D12	organic acid	250 mM Taurine	-0.43	-1.09	-0.87
41	F9	salt	125 mM Ammonium acetate	-0.38	-1.72	-4.45
42	H4	polymer	1.5% w/v Polyethylene glycol monomethyl ether 1,900	-0.3	-1.48	-3.51
43	D7	non detergent	500 mM Non Detergent Sulfobetaine 195 (NDSB-195)	-0.28	-1.65	-1.11
44	H6	polymer	1.5% w/v Polyethylene glycol 8,000	-0.3	-1.59	-3.7
45	F4	salt	125 mM Cesium chloride	-0.23	-1.11	-5.66
46	H5	polymer	1.5 % w/v Polyethylene glycol 3,350	-0.1	-1.33	-3.12
47	F5	salt	125 mM 4-Aminobutyric acid (GABA)	-0.01	-0.62	-
48	H7	polymer	1% w/v Polyvinylpyrrolidone K15	0.0	-1.4	-3.11
49	G4	salt	700 mM Lithium chloride	0.06	-0.44	-7.45
50	A4	amino acid/derivative	125 mM L-Arginine/125 mM L-Glutamic acid	0.18	-0.7	-2.23
51	A12	amino acid/derivative	250 mM 6-Aminohexanoic acid	0.20	-0.15	-0.73
52	A6	amino acid/derivative	250 mM L-Proline	0.35	-0.57	-1.51
53	E11	salt	250 mM Ammonium sulphate	0.35	-0.34	-5.05

54	D9	non detergent	500 mM Non Detergent Sulfobetaine 211 (NDSB-211)	0.53	-0.72	-0.63
55	A7	amino acid/derivative	60 mM L-Histidine	0.56	-0.42	-2.27
56	G1	salt	700 mM Potassium chloride	0.56	-0.28	-5.63
57	A9	amino acid/derivative	250 mM L-Serine	0.70	-0.22	-1.4
58	B9	osmolyte	250 mM Hydroxyectoine	0.88	-0.28	-2.22
59	E5	organic acid	2.5% v/v Tacsimate pH 7.0	0.76	-0.2	-3.95
60	F12	salt	500 mM Sodium chloride	-0.01	0.14	-5.41
61	F1	salt	250 mM Magnesium sulphate hydrate	0.52	0.39	-4.15
62	A8	amino acid/derivative	250 mM β-Alanine	0.74	0.1	-1.67
63	A5	amino acid/derivative	250 mM Glycine	0.80	0.33	-1.65
64	A3	amino acid/derivative	125 mM L-Arginine	0.91	0.06	-7.97
65	E4	organic acid	250 mM Succinic acid pH 7.0	1.25	1.48	-3.88
66	F7	salt	250 mM DL-Malic acid pH 7.0	1.50	1.51	-4.12
67	G2	salt	350 mM Sodium phosphate monobasic monohydrate/650 mM potassium phosphate dibasic	1.58	7.21	-4.74
68	E3	organic acid	250 mM Sodium malonate pH 7.0	1.62	1.83	-3.17
69	B11	osmolyte	1000 mM Methyl-α-D-glucopyranoside	1.78	0.64	-0.05
70	F8	salt	250 mM Lithium citrate tribasic tetrahydrate	2.42	2.63	-3.08
71	G3	salt	500 mM Sodium sulphate decahydrate	2.60	5.15	-4.8
72	B4	osmolyte	1250 mM Betaine monohydrate	1.66	0.62	2.48
73	B10	osmolyte	1250 mM Trimethylamine N-oxide dihydrate	3.06	3.13	1.31
74	G6	polyol and salt	20% v/v Glycerol/200 mM lithium chloride	3.53	1.66	0.3
75	B5	osmolyte	375 mM D-(+)-Trehalose dihydrate	3.73	2.73	1.14
76	G7	polyol	25% v/v Glycerol	4.36	2.5	2.42
77	B7	osmolyte	1000 mM D-Sorbitol	4.42	3.45	2.42
78	B6	osmolyte	1000 mM Xylitol	4.71	3.35	2.22
79	B8	osmolyte	1000 mM Sucrose	7.42	6.23	5.48
80	H1	polyol	25% v/v Polypropylene glycol P 400	8.5	-	-
81	H8	cyclodextrin	50 mM 6-O-α-D-Maltosyl-β-cyclodextrin	-	-	-
82	H9	cyclodextrin	5 mM (2-Hydroxypropyl)-β-cyclodextrin	-	-	-
83	H10	cyclodextrin	40 mM α-Cyclodextrin	-	-	-
84	H11	cyclodextrin	5 mM β-Cyclodextrin	-	-	-
85	H12	cyclodextrin	25 mM Methyl-β-cyclodextrin	-	-	-

86	C4	linker	250 mM Glutaric acid	-	-	-
87	C5	linker	40 mM Adipic acid	-	-	-
88	C11	chaotrope	15% w/v N-Methylformamide	-	-	-
89	C12	chaotrope	1.5% w/v Hypotaurine	-	-	-
90	D1	reducing agent	75 mM TCEP hydrochloride	-	-	-
91	D2	reducing agent	10 mM GSH (L-Glutathione reduced)/10 mM GSSG (L-Glutathione oxidized)	-	-	-
92	D8	non detergent	500 mM Non Detergent Sulfobetaine 201 (NDSB-201)	-	-	-
93	E2	organic acid	250 mM Oxalic acid dihydrate	-	-	-
94	F3	salt	125 mM Gadolinium(III) chloride hexahydrate	-	-	-
95	G12	polyol	25% v/v Formamide	-	-	-
96	A10	amino acid/derivative	250 mM L-Arginine ethyl ester dihydrochloride	-	-	-

Supplementary Table S2

Hydrogen bonds pairs and occupancy

Description of hydrogen bond pairs with more than 10% occupancy during 150 ns trajectories from 100 ns to 250 ns MD1 simulations and their respective occupancy at 100, 150, 200 and 250 ns.

	**galectin-4N/linker**			time (ns)			
MD 150 ns galectin-4	Donor	Acceptor	Occupancy (%)	100	150	200	250
	13TYR(HH)	175GLN(OE1)	57.5	60	81	32	100
	148ASN(D22)	171HIS(ND1)	56.1	49	58	60	100
	174GLN(E22)	9 TYR(O)	11.6	2	2	30	100
	175GLN(E22)	10 GLN(OE1)	11.3	17	6	11	0
	**galectin-4C/linker**			time (ns)			
	Donor	Acceptor	Occupancy (%)	100	150	200	250
	177ASN(H)	200GLY(O)	46.8	53	61	27	100
	178SER(H)	200GLY(O)	67.8	72	81	51	0
	181THR(HG1)	167PRO(O)	54.8	50	25	88	0
	183GLU(H)	167PRO(O)	29.6	37	48	4	100
	203THR(HG1)	175GLN(O)	51.2	71	38	45	100
	**galectin-4N/galectin-4C**			time (ns)			
	Donor	Acceptor	Occupancy (%)	100	150	200	250
	14ASN(D22)	207THR(OG1)	18.6	31	18	7	0
	36GLN(E22)	323ILE(OC1)	13.6	14	20	6	0
	211LYS(HZ3)	98 GLY(O)	34.2	40	38	24	0
	322GLN(E22)	101PHE(O)	68.8	69	78	59	100
MD 150 ns galectin-4-lactose	**galectin-4N/linker**			time (ns)			
	Donor	Acceptor	Occupancy (%)	100	150	200	250
	8GLY(H)	153GLN(OE1)	14.3	0	18	25	100
	13TYR(HH)	174GLN(OE1)	20.6	51	11	0	0
	13TYR(HH)	171HIS(O)	11.3	0	21	13	0
	148ASN(D22)	171HIS(ND1)	89	90	98	78	100
	156ARG(H)	8 GLY(O)	26.6	80	0	0	0
	156ARG(H12)	9 TYR(O)	23.6	67	4	0	0
	171HIS(HE2)	102GLU(OE1)	50.5	36	96	20	100
	**galectin-4C/linker**			Time (ns)			
	Donor	Acceptor	Occupancy (%)	100	150	200	250
	175GLN(E22)	183GLU(OE2)	19.6	2	20	36	0
	175GLN(E22)	183GLU(OE1)	17.6	7	23	22	0
	178SER(HG)	206ARG(NH1)	91.7	92	90	92	100
	178SER(HG)	180PRO(O)	96.3	94	97	97	100
	205ARG(HE)	173HIS(O)	73.8	73	74	75	0
	205ARG(H12)	177ASN(OD1)	10.3	9	21	1	0
	205ARG(H22)	177ASN(OD1)	53.2	46	54	60	100

	205ARG(H22)	176LEU(O)	26.2	27	35	17	0
	205ARG(H22)	173HIS(O)	44.2	45	34	54	100
	**galectin-4N/galectin-4C**			Time (ns)			
	Donor	Acceptor	Occupancy (%)	100	150	200	250
	36GLN(E22)	181THR(O)	65.8	43	92	62	100
	145GLN(E22)	183GLU(O)	44.9	68	41	26	0
	181THR(HG1)	102GLU(OE2)	70.8	67	91	53	100
	181THR(HG1)	102GLU(OE1)	34.2	32	13	58	0
	183GLU(H)	145GLN(OE1)	24.6	56	11	7	0

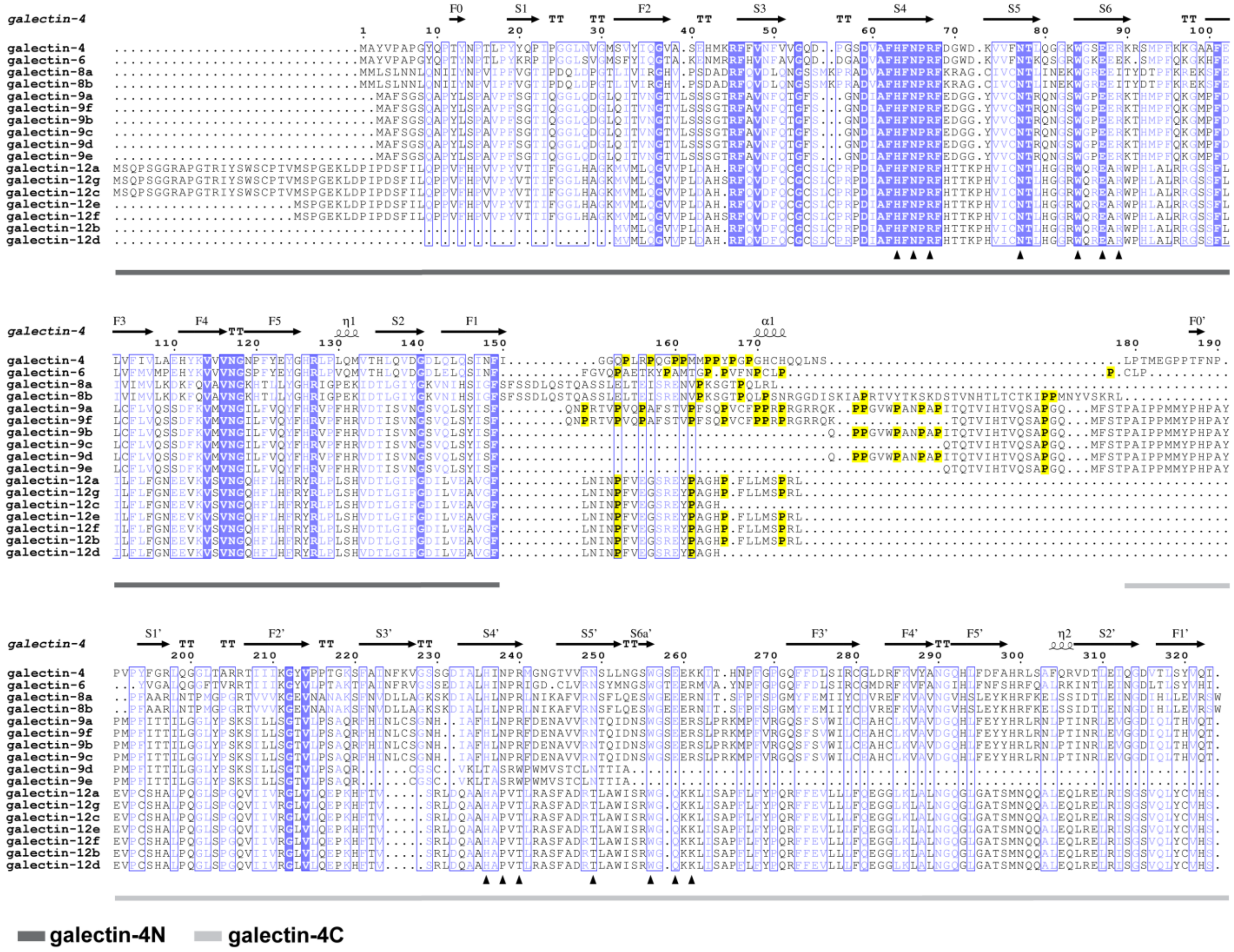

Supplementary Figure S1

Sequential alignment of the tandem-repeat galectins

Tandem-repeat galectins-6, -8, -9 and -12 and their respective isoforms were aligned against galectin-4 sequence. Marked with ▲ are the residues belonging to the carbohydrate-binding site. Galectin-4 secondary structure elements are placed above the alignment. Highlighted with a blue background and box are identical and similar residues, respectively. Marked with a yellow background are proline residues belonging to the linker-peptide regions.

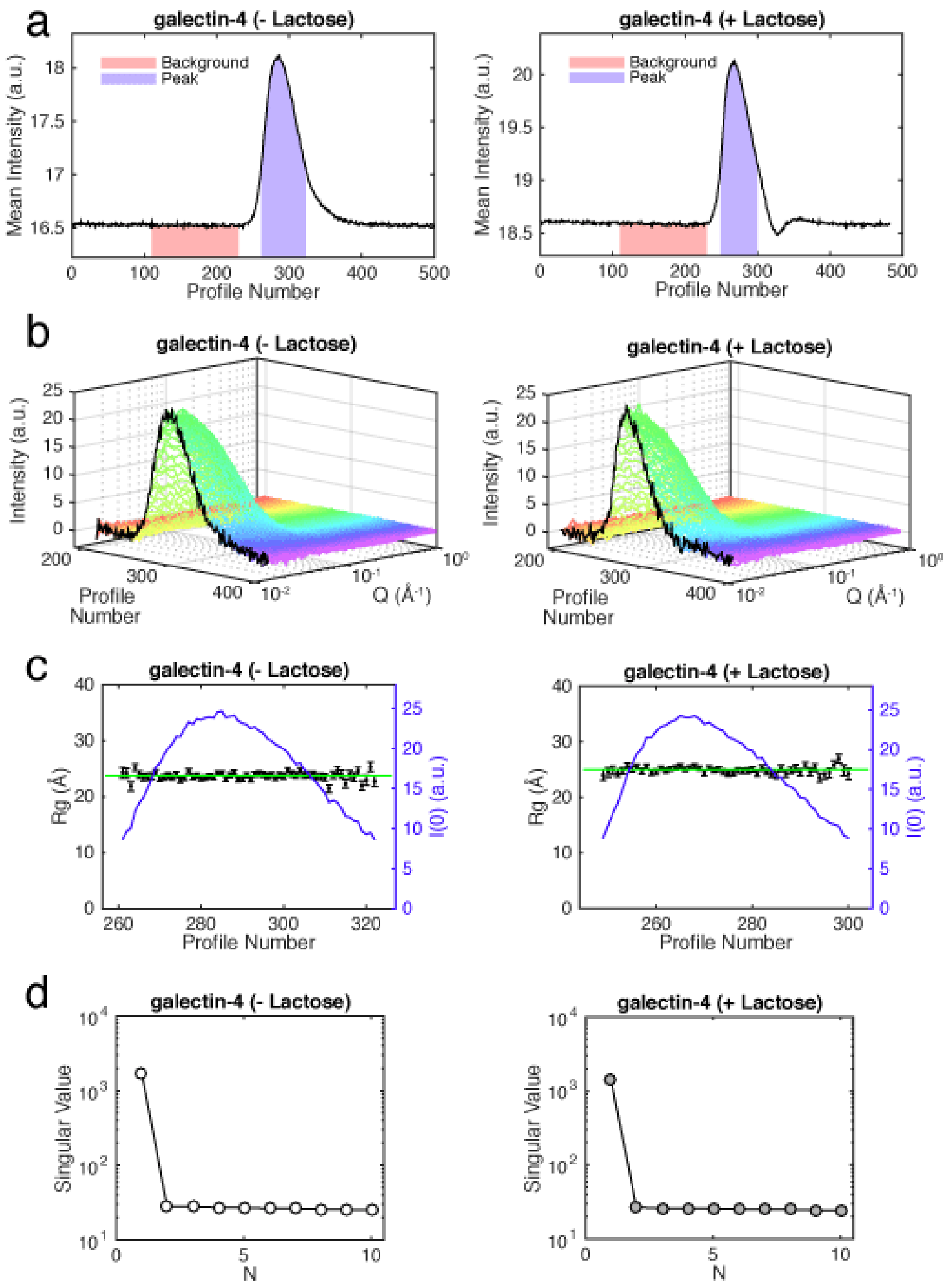

Supplementary Figure S2

Processing SEC-SAXS separations of galectin-4 in the presence and absence of lactose.

(a) SAXS/WAXS profiles corresponding to buffer background and sample were identified by examination of the mean x-ray intensity elution profiles. In the presence (+) and absence (-) of lactose, galectin-4 elutes as a single peak. (b) For each separation, the profiles in the background region were averaged together and subtracted from the remaining profiles. (c) The peak regions were analysed to verify their homogeneity before averaging. The radius of gyration (Rg) and forward scattering I(0) were found for each background-subtracted profile by Guinier analysis [1]. The apparent radius of gyration is constant through the peak (average value, solid green line) in both separations, showing the data to be free from concentration-dependent effects or incomplete separation that can sometimes be seen in SEC-SAXS data [2]. (d) Singular value decomposition (SVD) of each peak region identifies only one significant component, justifying the use of the average scattering profile for downstream analysis (see main text).

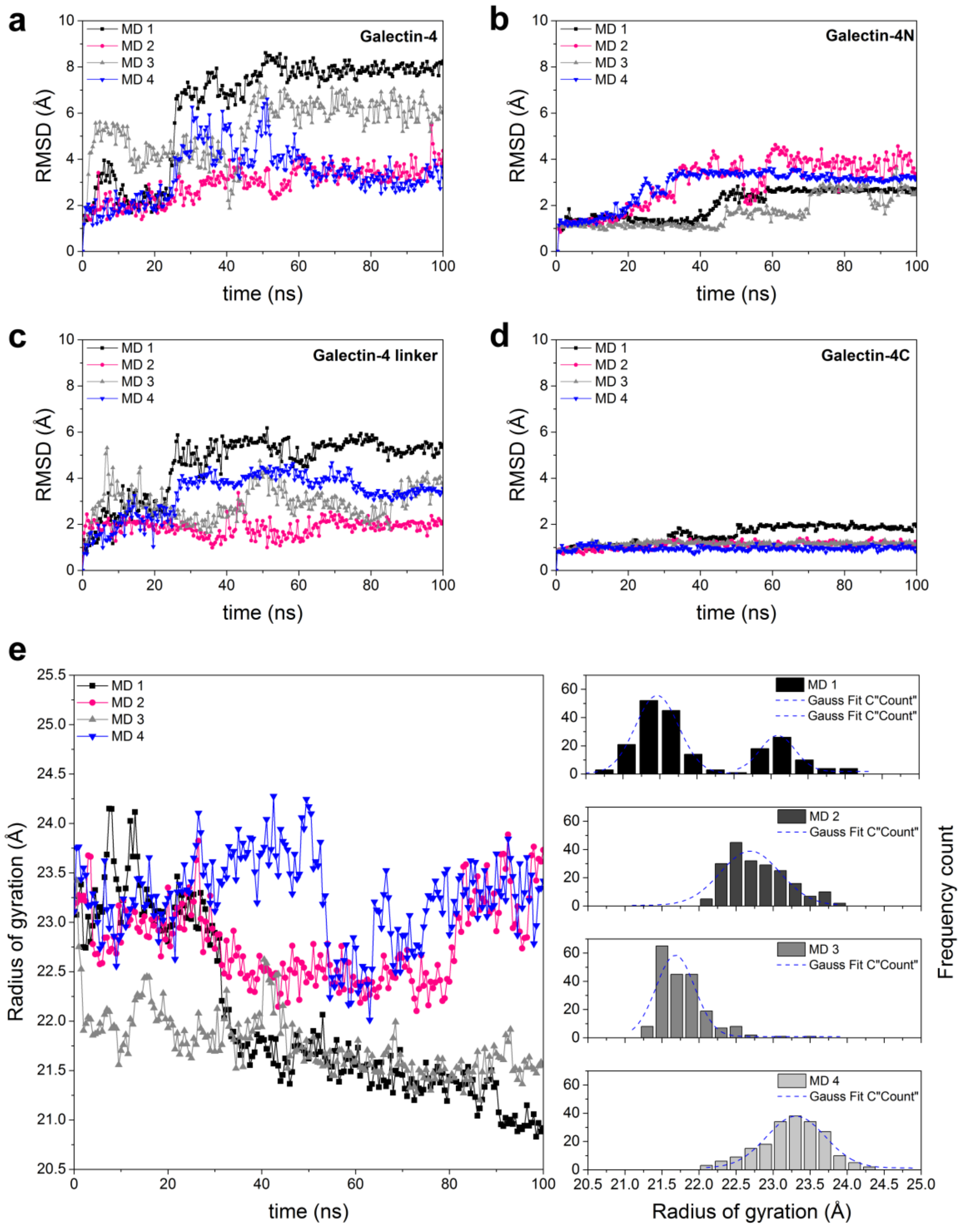

Supplementary Figure S3

RMSD plots and radius of gyration histogram for MD simulations without lactose

RMSD plots for MD1, MD2, MD3 and MD4 simulations without lactose, 100 ns trajectories. RMSD plot of backbone atoms (a) full-length galectin-4, (b) galectin-4N domain, (c) galectin-4 linker and (d) galectin-4C. (e) Radius of gyration and distribution of frequency plots for MD1, MD2, MD3 and MD4.

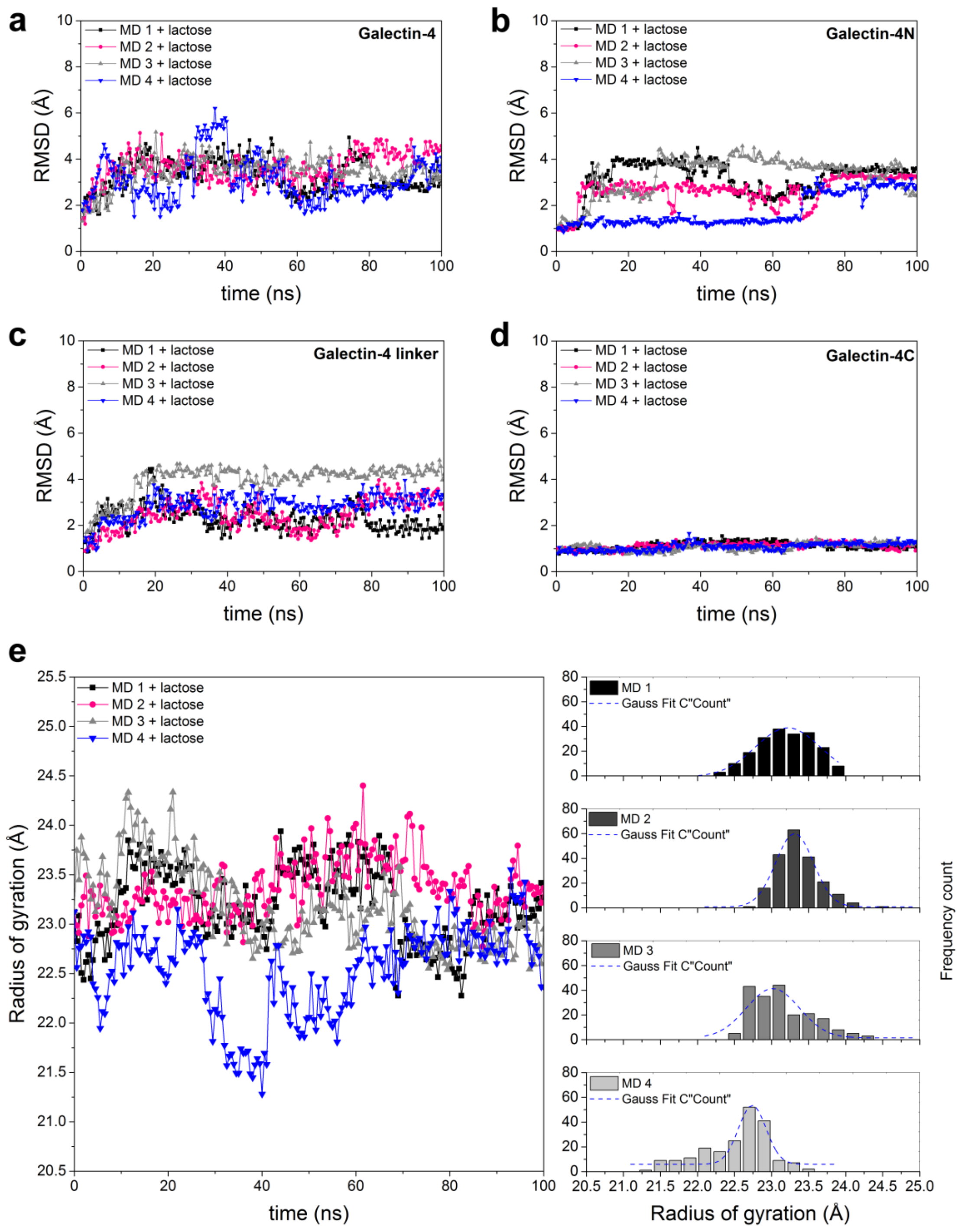

Supplementary Figure S4

RMSD plots and radius of gyration histogram for MD simulations with lactose

RMSD plots for MD1, MD2, MD3 and MD4 simulations with lactose, 100 ns trajectories. RMSD plot of backbone atoms (a) full-length galectin-4, (b) galectin-4N domain, (c) galectin-4 linker and (d) galectin-4C. (e) Radius of gyration and distribution of frequency plots for MD1, MD2, MD3 and MD4.

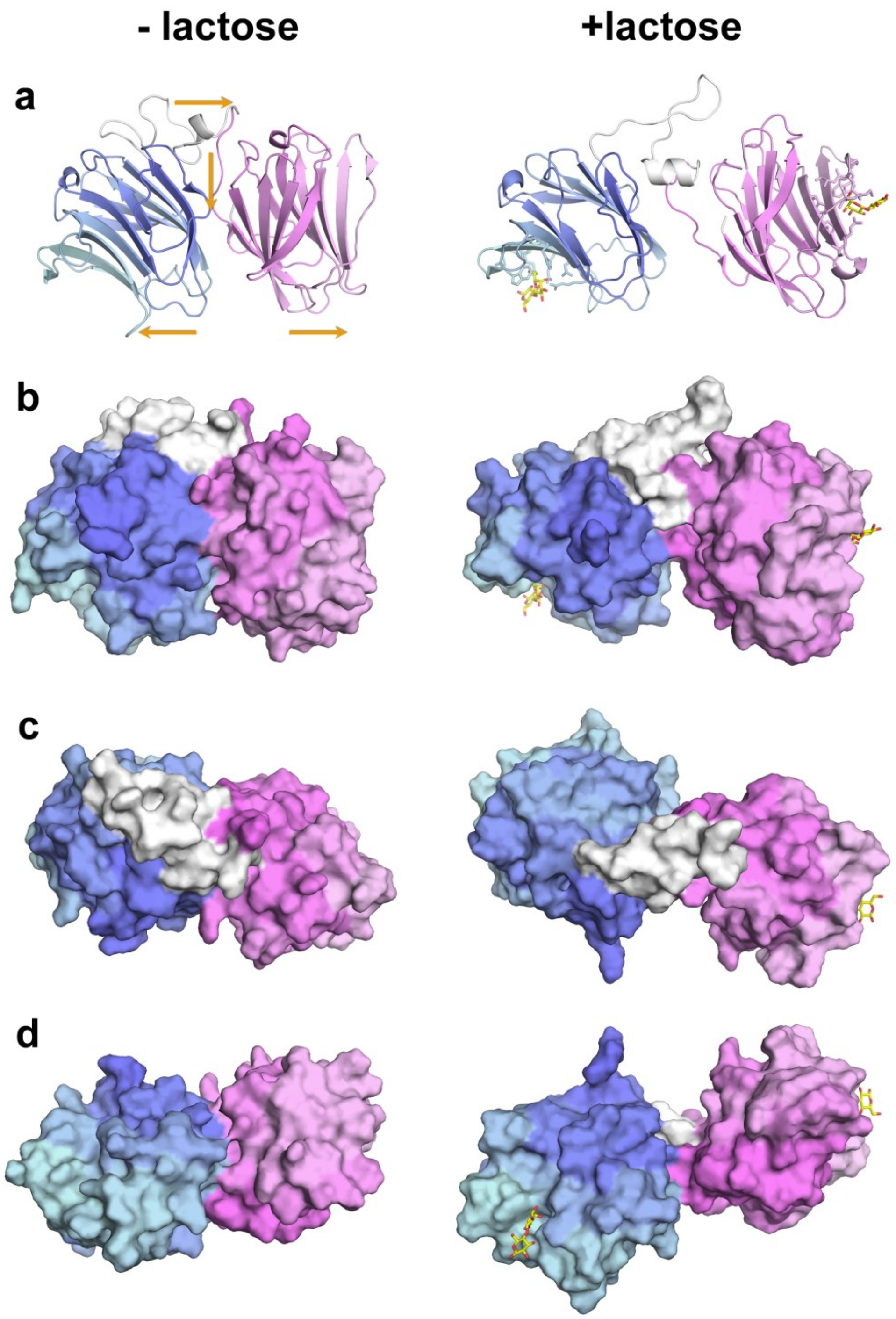

Supplementary Figure S5

Structure of galectin-4 without and with lactose at 250 ns

Structure of galectin-4 and galectin-4-lactose at the end of MD simulations. Represented in blue is galectin-4N domain, in white, the linker-peptide, in pink the galectin-4C domain and the lactose molecule in yellow. (a) Cartoon representation with front view from the structures; Surface representation of the (b) front view, (c) top view and (d) bottom view. Arrows indicate the tendency of the movement between domains for galectin-4 without and with lactose. Contact area between interfaces galectin-4N/linker, galectin-4N/galectin-4C and galectin-4C/linker correspond to 540 Å^2, 481 Å^2 and 334 Å^2 for galectin-4 without lactose and 325 Å^2, 202 Å^2 and 428 Å^2, for galectin-4 with lactose.

Supplementary References

1 A, G. & G, F. *Small-Angle scattering of x-rays*. (John Wiley & Sons, 1955).

2 Mathew, E., Mirza, A. & Menhart, N. Liquid-chromatography-coupled SAXS for accurate sizing of aggregating proteins. *J Synchrotron Radiat* **11**, 314-318, doi:10.1107/S0909049504014086 (2004).
